# Polymyxin B enhances acrosomal exocytosis triggered by calcium and the calcium ionophore A23187 in ejaculated boar spermatozoa

**DOI:** 10.1111/asj.13155

**Published:** 2019-04-11

**Authors:** Quzi S. Akter, Reza Rajabi‐Toustani, Kenji Shimizu, Yasushi Kuwahara, Tetsuma Murase

**Affiliations:** ^1^ The United Graduate School of Veterinary Sciences Gifu University Gifu Japan; ^2^ Department of Genetics and Animal Breeding Faculty of Animal Science and Veterinary Medicine Patuakhali Science and Technology University Barishal Bangladesh; ^3^ Fuji Nojo Service Fujinomiya Shizuoka Japan; ^4^ Laboratory of Veterinary Theriogenology Joint Department of Veterinary Medicine Faculty of Applied Biological Sciences Gifu University Gifu Japan

**Keywords:** acrosomal exocytosis, boar, calcium ionophore A23187, polymyxin B, sperm

## Abstract

Polymyxin B (PMB) is beneficial for boar semen storage since it neutralizes the endotoxin of bacteria. However, the direct effect of PMB on boar spermatozoa has been unknown. This study aimed to examine the effect of PMB on acrosomal exocytosis, an essential process for successful fertilization in boar spermatozoa. Ejaculated spermatozoa stored with BTS extender at 17°C were washed and incubated with 0–100 μM PMB for 20 min and then examined for % total motililty, vigor grade and viability. None of the parameters was significantly different between 0 and 50 μM PMB with a gradual decline at higher concentrations. Thus the effect on acrosomal exocytosis was investigated at 0–50 μM of PMB. Spermatozoa were preincubated with PMB for 10 min, incubated for stimulation of acrosomal exocytosis with Ca^2+^ and the calcium ionophore A23187 and then fixed with glutaraldehyde at 5, 10 and 15 min. Preincubation with PMB at 0.01–50 μM and 0.05–50 μM resulted in significant enhancement of acrosomal exocytosis at 10 min and 15 min of incubation, respectively. Preincubation with PMB followed by incubation without A23187 did not affect acrosomal exocytosis. These results suggest that PMB exerts effects on the acrosomal exocytosis triggered by Ca^2+^ and A23187 in boar spermatozoa.

## 1 INTRODUCTION

During the last decades, the use of artificial insemination (AI) in commercial pig herds by means of fresh diluted semen has increased considerably (Dominiek, Alfonso, Tom, Philip, & Ann, 2011). Diluted semen is stored at 15°C–20°C for several days until it used for AI (Huo, Yue, & Yang, 2002). Bacterial contamination is routinely observed in stored semen (Bryła & Trzcí, 2015), and Gram‐negative bacteria release an endotoxin, lipopolysaccharide (LPS) which decreases sperm motility (Okazaki et al., 2010). Polymyxin B (PMB) is a polycationic antibiotic and neutralizes endotoxic activity by binding to the LPS (Hosseinzadeh, Pacey, & Eley, 2003). Okazaki et al. (2010) reported that addition of PMB to stored boar spermatozoa is beneficial to attenuate decrease in motility.

Exocytosis of the acrosome, the so called acrosome reaction releases enzymes that allow spermatozoa to penetrate the oocyte vestments and thus is an essential process for fertilization (Yanagimachi, 1994). Acrosomal exocytosis is induced by physiological stimuli including progesterone and zona‐pellucida (Roldan, Murase, & Shi, 1994) but can be induced by the calcium ionophore A23187 (Murase, Imaeda, Kondoh, & Tsubota, 2004; Roldan & Harrison, 1989).

While PMB is beneficial for boar semen storage via antibacterial action, the direct effect of PMB, not via the neutralizing effect of PMB on bacteria, is totally unknown. Because acrosomal exocytosis is an essential step to fertilization, information on the direct effect is of significance to employ PMB for boar semen storage. This study investigated the effect of PMB on acrosomal exocytosis triggered by calcium and the calcium ionophore A23187 in boar spermatozoa that were freed from seminal plasma, extender, and hence from LPS. Since the direct effect of PMB on boar spermatozoa is totally unknown, its effect on motility and viability was first examined. Then the PMB concentrations found to be without effect on these traits were used to investigate the effect of PMB on acrosomal exocytosis in order to avoid false assessment by degenerative acrosome loss.

## 2 MATERIALS AND METHODS

### 2.1 Chemicals

Routine chemicals and reagents used in this study were of high purity grade and obtained from Sigma Chemical Co. (St. Louis, MO, USA) and FUJIFILM Wako Pure Chemical Corporation (Osaka, Japan) unless otherwise specified.

### 2.2 Media

Saline medium was used for washing and incubation of spermatozoa and consisted of 142 mM NaCl, 2.5 mM KOH, 10 mM glucose and 20 mM Hepes, adjusted to pH 7.55 at 20°C with NaOH (Roldan & Harrison, 1989). Sucrose medium containing 222 mM sucrose in place of NaCl in saline medium was used for washing spermatozoa. Saline and sucrose media also contained 0.1% (w/v) polyvinyl alcohol (average molecular weight of 30,000–70,000) and 0.1% (w/v) polyethylene glycol (consists of 2 moles of polyethylene glycol, 7000–9000). Saline medium contained 3 mM CaCl_2_ during sperm pre‐incubation and incubation for stimulation. A stock solution of 30 μM ionophore A23187 (Free acid; Calbiochem‐ Novabiochem/EMD Biosciences, La Jolla, CA, USA) was prepared in dimethyl sulfoxide (DMSO) and diluted in saline medium to give a final concentration of 0.3 μM. Polymixin B Sulfate (EMD Chemicals, San Diego, CA USA) was dissolved in H_2_O at 15 mM and kept frozen at −30°C as stock solution. For use in each experiment, a stock solution was allowed to thaw and then diluted with H_2_O for 100× stocks of 1, 2.5, 5, 7.5 and 10 mM. The 100× stocks were added to saline medium for the desired final concentrations.

### 2.3 Spermatozoa

Boar semen was purchased from Fuji Nojo Service, a porcine AI center in Japan. The sperm‐rich fraction was collected by the gloved‐hand method from three mature fertile boars (one Landrace and two Large White) aged 18–24 months and diluted using the Beltsville Thawing extender (Johnson, Aalbers, & Grooten, 1988) at a constant of 6‐fold and dispatched at 17°C to the laboratory, where semen was stored at 17°C for up to 3 days.

The experiments described here were approved by the Committee for Animal Research and Welfare of Gifu University.

Boar spermatozoa were washed according to Murase et al. (2004). Briefly, a portion of the stored semen was placed for sedimentation of large cell clumps by allowing it to stand for 10 min. The upper phase containing spermatozoa was overlaid onto a sucrose medium and centrifuged. The supernatant was aspirated and loose pellets of spermatozoa were mixed with the saline medium and then centrifuged and resuspended in the saline medium containing 3 mM CaCl_2_. Sperm concentration was adjusted to 2.4 × 10^7^ sperm/ml during preincubation (Experiment 2) and incubation (Experiments 1 and 2).

### 2.4 Experiment 1—Assessment of sperm charateristics

The washed spermatozoa were incubated with various concentrations of PMB (0, 10, 25, 50, 75 and 100 μM) at 37°C in air for 20 min. After incubation, spermatozoa were examined at 20× magnification under a phase contrast microscope at 38.5°C for subjective assessment of motile spermatozoa (% Total motility). Sperm viability was measured by staining with propidium iodide (PI) according to Harrison and Vickers (1990). Sperm vigor of flagellar beating was also subjectively evaluated on a grade of 0–4 (sperm vigor grade); 0, immotile; 1, sluggish movement; 2, movement with lowest speed; 3, sperm flagellum beating as fast as flagellum track is visible; and 4, spermatozoa beating flagellum as fast as unrecognizable track. An ejaculate from each boar was used on day 1, 2 and 3 of storage so that nine replicates (3 boars × 3 storage days) were carried out for each experiment.

### 2.5 Experiment 2—Induction of acrosomal exocytosis by calcium ionophore A23187

**Figure 1:**
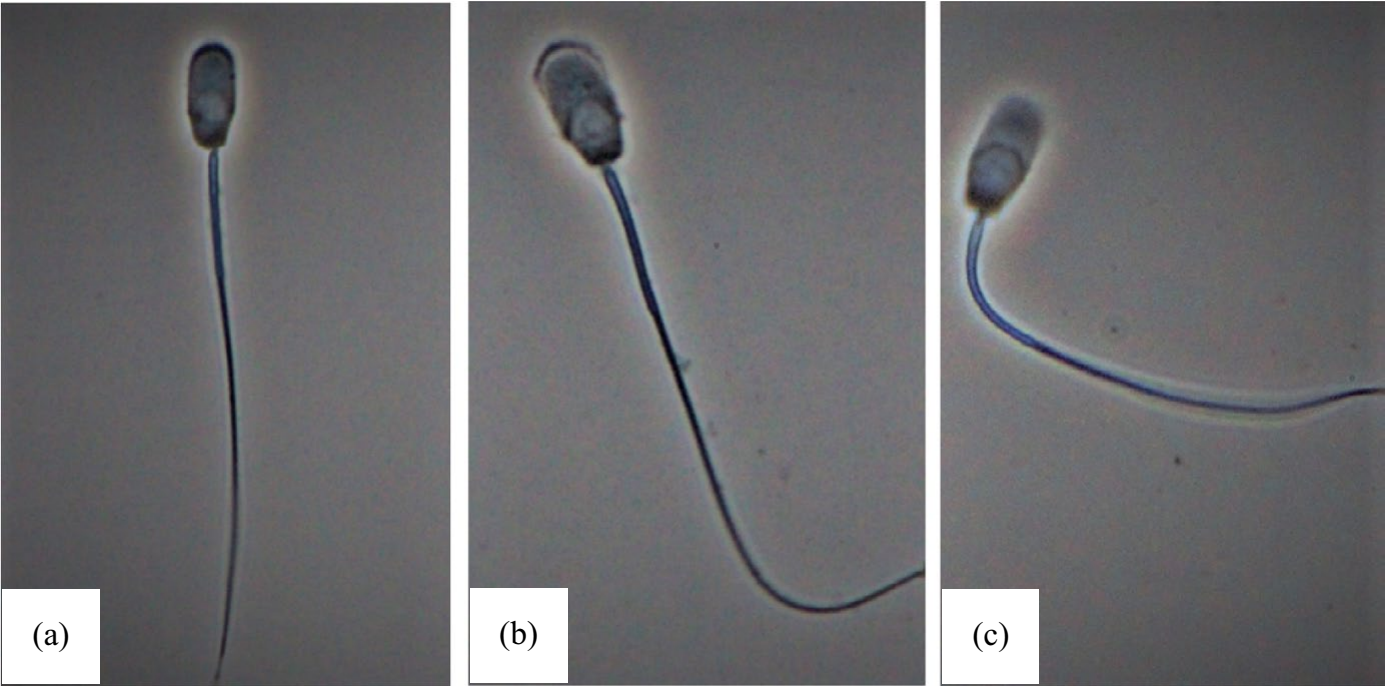
Photographs showing intact acrosome with dense apical ridge on the head (a), vesiculated acrosome with plasma and outer acrosomal membranes fused and remaining above the head (b) and complete loss of acrosome (c)

Washed spermatozoa from the three different boars were preincubated with various concentrations of PMB (0, 0.01, 0.05, 0.1 and 0.5 μM for the experiment with low concentrations of PMB and 0, 1, 10, 25 and 50 μM for the experiment with high concentrations of PMB) in saline medium at 37°C in air for 10 min and then incubated for stimulation with 0.3 μM A23187. H_2_O and DMSO were added as vehicle control for PMB (0 μM) and A23187, respectively. At various intervals of stimulation (5, 10 and 15 min), subsamples were taken and fixed by mixing with an equal volume of 2% glutaraldehyde/0.165 M cacodylate buffer (pH 7.3). A total of 200 spermatozoa were examined under a phase contrast microscope (400×) for the occurrence of acrosomal exocytosis according to Shams‐Borhan and Harrison (Shams‐Borhan & Harrison, 1981). Spermatozoa showing a head with a dense apical ridge were considered to be acrosome‐intact, and those showing vesiculated acrosome or acrosome completely lost were considered to have undergone acrosomal exocytosis (Figure 1). The percentage of spermatozoa displaying acrosomal exocytosis was obtained (% Acrosomal exocytosis).

### 2.6 Statistical analyses

All data are expressed as the mean ± standard error of the mean (*SEM*). % Total motility, % Viability and Sperm vigor grade were analysed using 2‐way ANOVA (storage time × PMB concentration) followed by Tukey's multiple comparison test except for % Viability by Fisher's protected least significant difference test. Percentage of acrosomal exocytosis was analysed using 2‐way ANOVA (time after stimulation × PMB concentration) followed by Bonferroni multiple comparison test. Values of *p* < 0.05 were considered to be statistically significant. All analyses were carried out using a statistical software program (GraphPad Prism Version 6.0; GraphPad Software, San Diego, CA, USA).

## 3 RESULTS

### 3.1 Experiment 1—Effect of PMB on sperm characteristics

Two‐way ANOVA did not reveal any significant main effect of storage nor interaction on any of the % Total motility, % Viability and sperm vigor grade but demonstrated a significant main effect of PMB on % Total motility, % Viability and sperm vigor grade (*p* < 0.0001). Since no significant main effect of storage was found, data were pooled across the days of storages (day 1, day 2 and day 3). For the parameters on which a significant main effect of PMB was found, values were compared among different concentrations of PMB.

**Figure 2:**
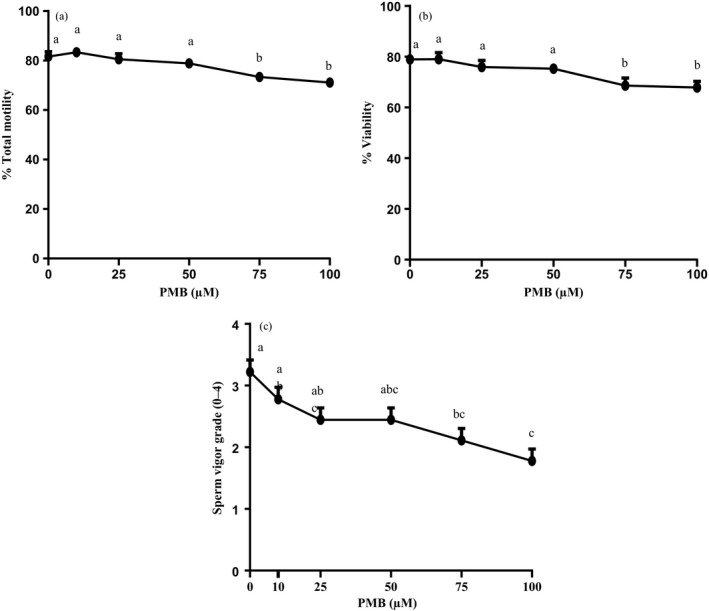
The effect of different concentrations of PMB (0–100 μM) on % Total motility (a), % Viability (b) and sperm vigor grade (c) of boar spermatozoa. The Main effect of PMB was significant for % Total motility, and % Viability (Two‐way ANOVA of storage days × PMB concentrations, *p* < 0.0001). a–e: Significant differences (*p* < 0.0001, respectively; Tukey's multiple comparison test except % Viability by Fisher's protected least significant difference test). Values are mean ± *SEM* from nine replicates (3 days × 3 boars)

Figure 2 shows % Total motility, % Viability and Sperm vigor grade in spermatozoa incubated with different concentrations of PMB. The % Total motility, Sperm vigor grade or Viability did not significantly differ up to 50 μM PMB with a significant decline at 75 and 100 μM (Figure 2a and c, Tukey's multiple comparison test, *p* < 0.0001; Figure 2b, Fisher's protected least significant difference test, *p* < 0.0001).

### 3.2 Experiment 2—Effect of PMB on acrosomal exocytosis

**Figure 3:**
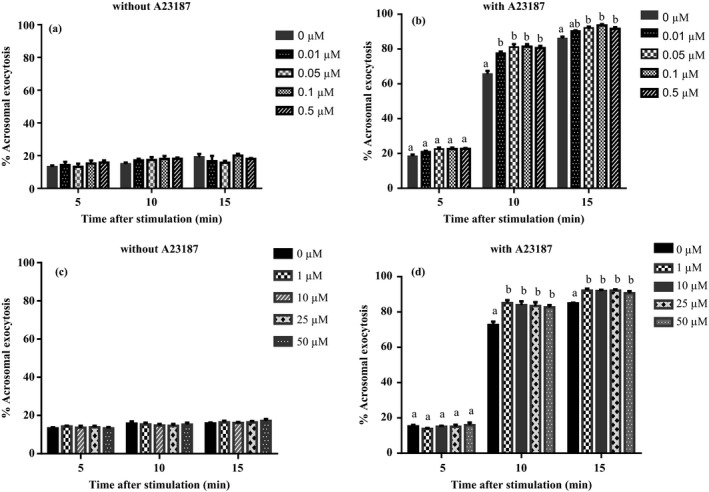
Effect of PMB on boar sperm acrosomal exocytosis induced by calcium and A23187. Acrosomal exocytosis after incubation in the absence (a and c) or presence (b and d) of 0.3 μM A23187 for 5, 10 and 15 min following preincubation for 10 min with different concentrations of PMB (a and b, 0–0.5 μM; c and d, 0–50 μM). Interaction between incubation time and concentrations of PMB was significant (Two‐way ANOVA,* p* < 0.0001). (a, b) Significant differences (Bonferroni's multiple comparison test; *p* < 0.05, respectively). Values are mean ± *SEM* from three replicates

Figure 3 shows % Acrosomal exocytosis in boar spermatozoa incubated for stimulation with A23187 following preincubation with PMB (Figure 3a and b: 0–0.5 μM; Figure 3c and d: 0–50 μM). In spermatozoa preincubated in the presence of PMB and subsequently incubated without A23187, there was no significant main effect of incubation time and PMB nor interaction (2‐way ANOVA, *p *>* *0.05, respectively; Figure 3a and c). When spermatozoa were stimulated with A23187, the two‐way ANOVA revealed that there was a significant interaction between incubation time and PMB (*p* < 0.0001). Preincubation with PMB at 0.01–50 μM and 0.05–50 μM resulted in significant enhancement of acrosomal exocytosis induced by A23187 at 10 min and 15 min of stimulation, respectively (Figure 3b and d).

## 4 DISCUSSION

The present study examined the effect of PMB on boar sperm acrosomal exocytosis in order to provide basic information for utilization of PMB as an additive to semen extender during boar semen storage. The results showed that PMB enhanced acrosomal exocytosis triggered by Ca^2+^ and A23187 at a concentration that did not affect sperm motility and viability suggesting that PMB can accelerate boar spermatozoa to undergo acrosomal exocytosis.

### 4.1 Effect on motility and viability

Unlike the results shown by Okazaki et al. (2010), in which sperm motility was maintained higher with PMB and penicillin G added during storage compared to penicillin G alone, we observed that after incubation with PMB, sperm motility decreased by 75–100 μM (Figure 2a). This discrepancy may be due to the washing of spermatozoa free from seminal plasma and the extender in this study. Spermatozoa in the extended semen may be exposed to less number of available PMB because of its binding to LPS while washed spermatozoa were directly exposed to high concentrations of PMB remaining available due to the absence of LPS in this study.

Treatment of boar spermatozoa with high concentrations of PMB (75 or 100 μM) resulted in the decrease in viability (Figure 2b). A recent study showed that addition of PMB to bull semen extender at 100 μg/ml (equal to ~75 μM) resulted in a beneficial effect on sperm motility after freezing‐thawing while a higher concentration (1,000 μg/ml, equal to ~750 μM) of PMB reduced viability (Rashedi, Fazeli, Gholami, & Bahreini, 2017). This indicates that the antibiotic is spermicidal depending on the concentrations used. However, it should be taken into consideration that PMB molecules that bind to LPS are expected to be no longer available to spermatozoa and that thus depending on the LPS content in semen, the direct effect of PMB on spermatozoa may be decreased.

### 4.2 Effect on acrosomal exocytosis

It may be possible that PMB exerted its effect during preincubation followed by enhancement of acrosomal exocytosis and/or that PMB entered spermatozoa and stayed inactive until spermatozoa were stimulated with Ca^2+^ and A23187. The former possibility implies that during preincubation, PMB capacitated spermatozoa at least partially and facilitated the induction of acrosmal exocytosis upon stimulation. When PMB was used as protein kinase C inhibitor in somatic cells, the cells were preincubated, for instance, 30 min prior to stimulation with an agonist (Li, Zhang, Zhang, Zhou, & Lin, 2016; Wallin et al., 2003) so that the cells could incorporate PMB during the preincubation period. In fact, PMB has been shown to be internalized into somatic cells at as low concentrations as nM levels via caveolae‐mediated pathway (Hamill, McCoy, Wexselblatt, Esko, & Tor, 2016) while the presence of caveolin‐1, a major protein that constitutes caveolae has been demonstrated in boar spermatozoa (van Gestel et al., 2005). Thus these reports support the latter possibility.

Although PMB is known to be a modulator of signaling molecules in somatic cells and inhibits a variety of enzymes such as protein kinase C (Li et al., 2016; Mizumaki et al., 2002; Radallah, Nogaro, & Fournier, 1999; Wallin et al., 2003), a phospholipid‐sensitive Ca^2+^ protein kinase (Mazzei, Katoh, & Kuo, 1982), calmodulin (CaM) (Hegemann, van Rooijen, Traber, & Schmidt, 1991), CaM‐dependent phosphodiesterase from bovine heart in vitro (Hegemann et al., 1991), Ca^2+^/CaM‐dependent protein kinases (Depaoli‐Roach, Roach, & Larnerf, 1979; Walsh, Vallet, Autric, & Demaille, 1979), K^+^‐ATP channels (Harding, Jaggar, Squires, & Dunne, 1994), Ca^2+^‐ATPase, p‐nitrophenyl phosphatase and phosphorylase kinase of rabbit skeletal muscle sarcoplasmic reticulum membranes (Ktenas, Sotiroudis, & Evangelopoulos, 1989), the mechanisms by which acrosomal exoycytosis was enhanced by PMB were not clear in the present study. Further investigations are required to clarify the mechanisms.

This study sought a possible direct effect of PMB on boar spermatozoa freed from seminal plasma and extender and hence from LPS because PMB is known to act as an inhibitor of a variety of signaling molecules in somatic cells. The results showed that PMB decreased motility and viability at μM levels but increased acrosomal exocytosis at lower concentrations. Thus the study suggests that PMB has direct effects on boar spermatozoa. This should be taken into consideration when it is added to boar semen extender. In our previous study, boar spermatozoa collected during the summer season, when “summer infertility” takes place, were found to be more sensitive to Ca^2+^ and A23187 suggesting that boar spermatozoa have a higher inducibility of acrosomal exocytosis in summer (Murase, Imaeda, Yamada, & Miyazawa, 2007). It may be important to point out that if PMB is added to semen diluted in summer, the inducibility of spermatozoa would be further increased. On the contrary, it is possible that PMB could increase the acrosomal exocytosis of spermatozoa that may have decreased their ability to undergo acrosomal exocytosis for some reason, for example, by sub‐fertility. It is still unknown whether PMB similarly enhances acrosomal exocytosis when it is added to extended semen during storage and whether the increased inducibility of acrosomal exocytosis is related to fertility after AI. Further experiments are required to address these issues.

## 5 CONCLUSIONS

The present study showed that PMB exerts effect on sperm function as revealed by acrosomal exocytosis in boar spermatozoa and pointed out the importance to take into consideration the effect of PMB on acrosomal exocytosis in boar spermatozoa, when it is used for AI in pigs. Further studies are required to clarify the mechanisms by which polymyxin B acts on acrosomal exocytosis in spermatozoa and to reveal the effect of PMB added to diluted semen during storage on acrosomal exocytosis.
